# Pneumomediastinum complicating COVID-19: a case series

**DOI:** 10.1186/s40001-021-00585-9

**Published:** 2021-09-26

**Authors:** Sérgio Henrique Bastos Damous, Jones Pessoa dos Santos Junior, Álvaro Vicente Alvarez Pezzano, Mohamad Abdul Majid Chams, Nathaly Haritov, Ricardo Waksman, Helber Vidal Gadelha Lima, Jocielle dos Santos Miranda, Roberto Rasslan, Edivaldo Massazo Utiyama

**Affiliations:** Division of General Surgery and Trauma, Department of Surgery, Hospital das Clínicas da Faculdade de Medicina da Universidade de São Paulo (HC/FMUSP), Dr. Enéas de Carvalho Aguiar Av. 255. Cerqueira Cesar, São Paulo, 05402-000 Brazil

**Keywords:** COVID-19, SARS-CoV-2, Pneumomediastinum

## Abstract

**Background:**

Pneumomediastinum is a rare complication of COVID-19 pneumonia, which may or may not be associated with invasive ventilatory support. Therefore, the report and findings associated with its evolution can be of great contribution in the management of this unknown disease.

**Case presentation:**

Here, we present a series of four patients with severe pneumomediastinum requiring intensive care unit. These patients developed pneumomediastinum before or during orotracheal intubation (OTI) or without OTI. The four patients were three men and one woman with a mean age of 60.5 years (48–74 years). No patients had a known history of lung disease or traumatic events, except for one patient who had a history of smoking, but who was without parenchymal disease. All intubations were performed without complications. No cases of pneumomediastinum occurred after tracheostomy, and none of the patients had tomographic or bronchoscopic evidence of tracheal injury. Although the pneumomediastinum observed in our cases was apparently not related to a violation of the aerodigestive track, this complication was associated with a worse prognosis.

**Conclusion:**

Pneumomediastinum is a rare complication of COVID-19 pneumonia, and the most likely etiopathogenesis is severe pulmonary involvement, which may or may not be associated with invasive ventilatory support. Future studies with a greater number of cases should elucidate the relationship of pneumomediastinum to a probable prognostic factor.

## Background

Pneumomediastinum is a frequent sign of clinical concern with potentially threatening consequences [1]. It can be classified as primary pneumomediastinum, which is also called spontaneous pneumomediastinum and is defined as the presence of air in the mediastinum without any defined cause [2]; secondary pneumomediastinum develops as a consequence of a distinct underlying pathology or thoracic injury, resulting in intrathoracic dissection of air through the mediastinal planes [1].

 The lungs of patients with COVID-19 have significant interstitial involvement with edema, protein exudates, vascular congestion and inflammatory changes with low compliance and reduced elastance [3]. Therefore, when there is a pressure gradient between the alveoli and the pulmonary interstitium in the fibrotic and hypoelastic lung, alveolar rupture and the consequent leakage of air into the interstitium can occur. Due to the pressure gradient between the pulmonary periphery and the mediastinum, the air present in the pulmonary interstitium flows toward the pulmonary hilum and the mediastinum [4].

 The Hospital das Clínicas is the largest public tertiary hospital in the state of São Paulo, Brazil. It has approximately 900 beds, with approximately 300 intensive care beds. In the context of the COVID-19 pandemic, the hospital was entirely dedicated to the care of COVID-19 as the State’s main referral center for serious cases. Here, we report a series of four cases of patients diagnosed with COVID-19 who developed pneumomediastinum.

## Case series

We present the cases of four patients with COVID-19 disease verified through nasotracheal SARS-CoV-2 PCR or chest computerized tomography (CT) who were referred to our department from April to July 2020. We recorded the baseline patient characteristics, including comorbidities, ventilation data, information regarding the signs of pneumomediastinum and its diagnosis, ongoing management and outcome. Consent for the use of anonymized data and imaging was obtained from the next of kin.

These patients developed pneumomediastinum before or during orotracheal intubation (OTI) or without OTI. The data are summarized in Table 1. The four patients were three men and one woman with a mean age of 60.5 years (48–74 years). No patients had a known history of lung disease or traumatic events, except for one patient who had a history of smoking but who was without parenchymal disease. All intubations were performed without complications. No cases of pneumomediastinum occurred after tracheostomy, and none of the patients had tomographic or bronchoscopic evidence of tracheal injury.

**Table 1:** Case series of patients who developed pneumomediastinum during COVID-19 infection

	Age	Comorbidities	SARS-CoV-2 PCR	Lung severity score on chest CT scans (%)	Pneumomediastinum	Oro-tracheal intubation	PEEP (cmH_2_O)	Bronchoscopy
Case 1	50	Chronic arterial hypertension and kidney transplantation	Negative	> 50	10th day	No	–	No
Case 2	70	Ex-smoker and diabetic	Positive	> 50	18th day	Yes	8–10	Yes
Case 3	48	None	Positive	> 50	14th day	Yes	8–10	Yes
Case 4	74	Mastectomy	Positive	> 50	20th day	Yes	8–10	No

*CT* computerized tomography

### Case 1

**Fig. 1:**
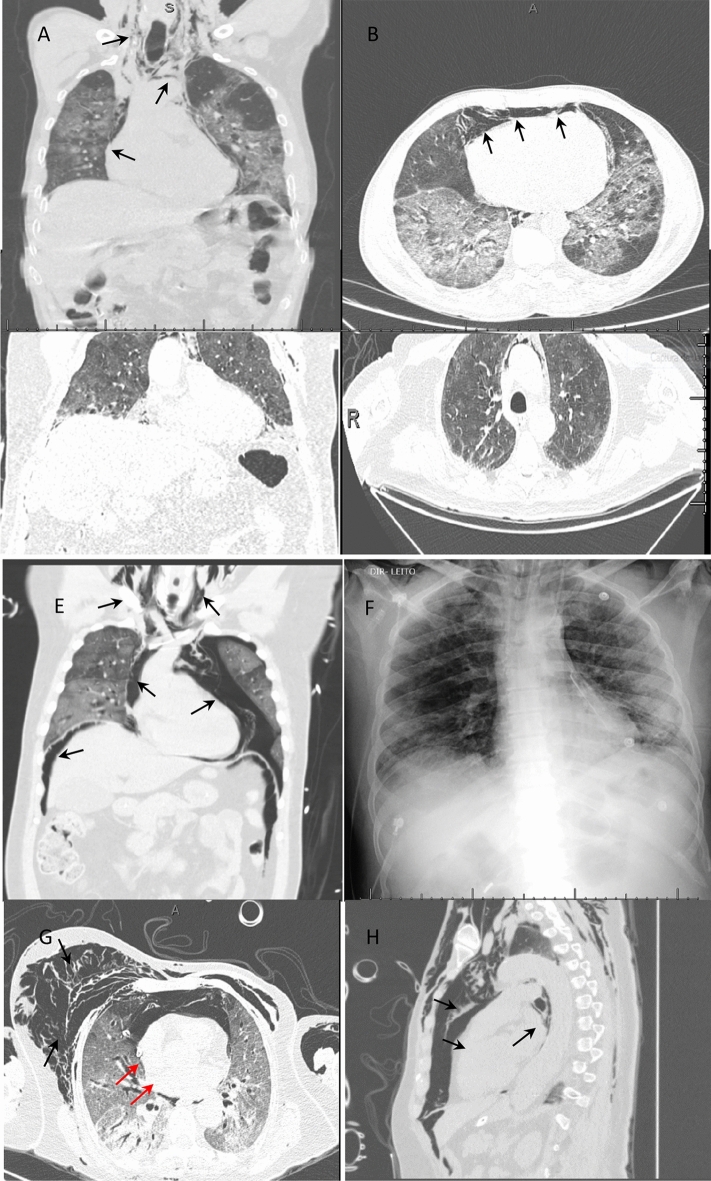
Chest CT scans or radiograph of the chest showing pneumomediastinum (black arrows). **A**, **B** Case 1. **C**, **D** Case 2. **E**, **F** Case 3. **G**, **H** Case 4. Red arrows: chest drainage

A 50-year-old man was admitted to our hospital after transfer from a basic health unit with a chest CT compatible with viral infection by SARS-CoV-2. He had cough, respiratory distress, myalgia, fever and episodes of diarrhea for 10 days, and he was using ceftriaxone and clarithromycin with oxygen support. In his medical history, the patient reported tabagism, systemic hypertension, a kidney transplant 6 years previously, and the use of tacrolimus, everolimus and prednisone. In our service, we request laboratory tests, including a nasopharyngeal swab PCR test, to confirm infection with SARS-CoV-2 and perform an additional chest CT. Although the admission test was negative for SARS-CoV-2, chest CT showed multiple ground-glass pulmonary opacities with scattered foci of consolidation, which were multifocal and bilateral with an estimated extent greater than 50% [5, 6] that were compatible with viral pneumonia, in addition to extensive anterior, middle and posterior pneumomediastinum dissecting the bilateral anterior cervical plane (Fig. 1A, B). Soon after tomography was performed, there was rapid deterioration of the respiratory condition requiring orotracheal intubation (OTI). In the intensive care unit, piperacillin  +  tazobactam, vancomycin, oseltamivir and corticosteroids were introduced. During disease progression, there was clinical worsening with hemodynamic instability, and it was not possible to perform complementary exams, such as upper digestive endoscopy and bronchoscopy. Over the next 10 days, the patient was maintained on high doses of vasoactive drugs (norepinephrine and vasopressin) and suffered from multiple system dysfunction that resulted in death.

### Case 2

A 70-year-old man who was diabetic was admitted to our hospital on the 13th day after the onset of flu-like symptoms with invasive ventilatory support and chest CT showing suspected infection by SARS-CoV-2. Ceftriaxone, azithromycin, heparin and oseltamivir were used. He was directed to the intensive care unit, his antibiotics were changed (piperacillin/tazobactam and vancomycin), and support was started with vasoactive drugs. The nasopharyngeal swab PCR test was positive. On the 18th day, the patient was placed in a prone position as an adjuvant therapy for improving ventilation.

After a favorable response, it was decided to suspend the neuromuscular block, decrease sedation and decrease the parameters of the invasive mechanical ventilation. After 24 h, the patient presented subcutaneous emphysema in the cervical and thoracic regions without worsening of the ventilatory parameters. On chest CT, there was extensive subcutaneous emphysema and pneumomediastinum with multiple ground-glass pulmonary opacities associated with thickening of the interlobular septa with an estimated extent greater than 50% (visual analysis) [5, 6] (Fig. 1C, D). During disease progression, bronchoscopy and tracheostomy were performed without complications. After 28 days in the intensive care unit with treatment with tigecycline for carbapenem-resistant *Klebsiella pneumoniae* in the tracheal secretions, the patient presented with hemodynamic worsening, which progressed to death.

### Case 3

A 48-year-old man without comorbidities was admitted to our service on mechanical ventilation 12 days after the onset of flu-like symptoms. Intubation without complications by another service was reported. The test for SARS-CoV-2 (nasopharyngeal swab PCR) was positive. Ceftriaxone and prophylactic anticoagulation were started. On the 2nd day after admission, the patient presented subcutaneous emphysema on chest CT, showing massive emphysema in the anterior and posterior cervical regions and chest wall with pneumomediastinum, left nonhypertensive pneumothorax and pneumoperitoneum, and multiple ground-glass opacities that were multifocal with consolidation and an extent greater than 50% (visual area) [5, 6] (Fig. 1E, F). Thoracic drainage was performed on the left lung with a pigtail drain without complications, and bronchoscopy was performed without evidence of airway injury. During progression, the patient underwent percutaneous tracheostomy and treatment with meropenem  +  vancomycin. He is currently undergoing home rehabilitation without the need for oxygen therapy.

### Case 4

A 74-year-old female patient with a history of left mastectomy for malignant breast cancer was undergoing adjuvant chemotherapy with paclitaxel. There was no pulmonary or pleural metastasis. She was admitted to our service after 17 days of progression of the symptoms of cough, myalgia and dyspnea. The nasopharyngeal swab PCR test for SARS-CoV-2 showed a positive result. She was given piperacillin  +  tazobactam and prophylactic heparin, and azithromycin was added. On the 3rd day of hospitalization, the patient progressed with deterioration of respiratory function requiring orotracheal intubation, which was performed without complications, and she was referred to the intensive care unit. Eight hours after OTI, the patient developed extensive subcutaneous emphysema and decreased auscultation in the right chest, with suspicion of pneumothorax and right chest drainage. She was placed on mechanical ventilation, which was volume-controlled, with a PEEP of 7 (cmH_2_O), an FiO_2_ of 80% and a SatO_2_ of 97%. Chest CT showed multiple ground-glass pulmonary opacities, which were sometimes associated with thickening of the interlobular septa and fine reticulate between consolidations, and presented a diffuse distribution with an estimated extent of pulmonary involvement on tomography greater than 50% (visual analysis) [5, 6]. Soft tissue emphysema was observed in the bilateral chest wall with extension into the superficial and deep planes of the cervical region and extensive pneumomediastinum. A thoracic drain was placed on the right side with the end in the medial region of the hemithorax (Fig. 1G, H). The patient progressed to refractory shock, which was not directly related to pneumothorax or pneumomediastinum. The antimicrobial regimen was expanded by introducing meropenem and vancomycin, and hemodynamic support was optimized. Despite all measures, the patient progressed to multiple organ dysfunction 48 h after OTI. Bronchoscopy was not performed due to the patient’s clinical condition, but there was no evidence of tracheal injury on tomography.

## Discussion

Due to the COVID-19 pandemic, there are currently reports of pneumomediastinum as a rare complication of COVID-19 pneumonia. Pneumomediastinum could result directly from the pathogenesis of SARS-CoV-2 (rupture of pulmonary bullae) or secondary to intensive care management due to airway trauma during tracheal intubation, barotraumas or repositioning maneuvers [7–9].

Here, four cases of pneumomediastinum complicating COVID-19 pneumonia were presented. We did not find that this complication was due to iatrogenic causes. Although two of the patients were not subjected to fiberoptic bronchoscopy due to their clinical instability, it was not possible to identify any airway discontinuity by computed tomography. The patient in case 1 had not even been intubated when he presented with pneumomediastinum. Additionally, the other patients maintained a low positive end-expiration pressure of 8–10 cmH_2_O.

All cases presented with more than 10 days of disease, and more than 50% showed pulmonary involvement by chest CT, which evolved into severe dyspnea with a PEEP between 8 and 10 and had an unfavorable outcome (death). It was not possible to correlate pneumothorax with pneumomediastinum or OTI as a possible cause, as there was no difficulty in terms of OTI. It is assumed, then, that pneumomediastinum must have been secondary to the pulmonary deterioration caused by COVID-19, including the formation of pulmonary fibrosis and the rupture of bubbles in the mediastinum. The patients in cases 3 and 4 even had evidence of extraperitoneal air dissection, as shown in Fig. 1. We hypothesized that reactive pleural thickening associated with COVID-19 prevents air from spreading to the pleural space; therefore, some cases do not show evidence of pneumothorax [7, 10].

Kong et al. [11] reported a case of pneumomediastinum in an elderly patient after 14 days of illness, and it was not possible to determine any causal event, similar to our cases. Unlike the patients in our case series, this patient evolved satisfactorily with conservative treatment. Pneumomediastinum was a chest CT finding in a 23-year-old patient who was totally asymptomatic, with no emphysema or pneumothorax, and 7 days later, this patient showed complete resolution of the pneumomediastinum [9]. 

There have been two other reports of pneumomediastinum in young patients with COVID-19 without previous illnesses or traumas, who presented completely different courses of disease. The first patient, who was 37 years old, developed mediastinal emphysema and pneumothorax. No abnormalities, such as a small bulla or emphysema, were observed in the initial chest CT, which was performed while the patient was receiving noninvasive mechanical ventilation in the intensive care unit. The authors suggest that mediastinal emphysema results from a sudden increase in alveolar pressure, causing alveolar rupture and air leakage with interstitial emphysema. The patient recovered with oxygen therapy [8]. In the other case, a 36-year-old patient succumbed to illness after 2 days due to respiratory failure and acute respiratory distress despite supportive care [7]. This second case is similar to the cases in our case series.

In another case series, five patients developed pneumomediastinum 4 h–14 days after tracheal intubation. All patients were invasively ventilated due to respiratory compromise with severe hypoxemia despite receiving maximal ward-based oxygen therapy. One patient developed pneumomediastinum immediately following prone positioning maneuvers [12]. In these cases, pneumomediastinum was secondary to barotrauma or repositioning maneuvers, unlike that observed in our series, in which it was not possible to associate pneumomediastinum with a causal factor besides COVID-19 infection.

Although the pneumomediastinum observed in our cases was apparently not related to a violation of the aerodigestive track, this complication was associated with a worse prognosis [7]. Although death does not appear to be directly related to pneumomediastinum in most cases, three of the four patients died. This must indicate that they were in an advanced phase of COVID-19.

## Conclusion

Pneumomediastinum is a rare complication of COVID-19 pneumonia, and the most likely etiopathogenesis is severe pulmonary involvement, which may or may not be associated with invasive ventilatory support. Future studies with a greater number of cases should elucidate the relationship of pneumomediastinum to a probable prognostic factor.

## Data Availability

The dataset supporting the conclusions of this article is included within the article.

## Abbreviations

OTI: Oro-tracheal intubation
CT: Computerized tomography
